# The Rough Guide to Monocytes in Malaria Infection

**DOI:** 10.3389/fimmu.2018.02888

**Published:** 2018-12-07

**Authors:** Amaya Ortega-Pajares, Stephen J. Rogerson

**Affiliations:** Department of Medicine at Royal Melbourne Hospital, Peter Doherty Institute for Infection and Immunity, University of Melbourne, Melbourne, VIC, Australia

**Keywords:** leukocytes, innate immunity, plasmodium, trained immunity, cytokine, phagocytosis

## Abstract

While half of the world's population is at risk of malaria, the most vulnerable are still children under five, pregnant women and returning travelers. Anopheles mosquitoes transmit malaria parasites to the human host; but how *Plasmodium* interact with the innate immune system remains largely unexplored. The most recent advances prove that monocytes are a key component to control parasite burden and to protect host from disease. Monocytes' protective roles include phagocytosis, cytokine production and antigen presentation. However, monocytes can be involved in pathogenesis and drive inflammation and sequestration of infected red blood cells in organs such as the brain, placenta or lungs by secreting cytokines that upregulate expression of endothelial adhesion receptors. *Plasmodium* DNA, hemozoin or extracellular vesicles can impair the function of monocytes. With time, reinfections with *Plasmodium* change the relative proportion of monocyte subsets and their physical properties. These changes relate to clinical outcomes and might constitute informative biomarkers of immunity. More importantly, at the molecular level, transcriptional, metabolic or epigenetic changes can “prime” monocytes to alter their responses in future encounters with *Plasmodium*. This mechanism, known as trained immunity, challenges the traditional view of monocytes as a component of the immune system that lacks memory. Overall, this rough guide serves as an update reviewing the advances made during the past 5 years on understanding the role of monocytes in innate immunity to malaria.

## Introduction

Malaria is a parasitic disease mostly present in poor tropical and subtropical countries. In 2016 alone, malaria accounted for 445,000 deaths and 216 million clinical episodes (1). When an infected female Anopheles mosquito feeds on human blood, she injects sporozoites, motile forms of the *Plasmodium* parasite, that travel to the liver. Within the hepatic cells, parasites divide to form schizonts that rupture and release merozoites into the bloodstream where they infect erythrocytes. The cycle of parasite division and merozoite invasion of new RBCs coincides with the clinical symptoms of malaria illness, which include fever, chills and headaches. The clinical symptoms progress from asymptomatic infection to uncomplicated disease to severe malaria to death. Life-threatening malaria occurs when infection leads to dysfunction of organs including the brain, placenta, kidney or lungs, or causes abnormalities in the patient's blood or metabolism, such as anemia.

Since sporozoites (the infective form) rapidly leave the skin (2), little is known about how skin innate immune cells interact with them. During blood stage infection, monocytes control parasite burden and contribute to host protection through phagocytosis, cytokine production and antigen presentation, but they also drive inflammation and sequestration of infected red blood cells (iRBCs) in organs (such as the brain, placenta, or lungs). Monocytes come in different “flavors” [discussed in (3). According to the levels of CD14 and CD16 expressed on their surface, they are classified in three subsets: classical or inflammatory (CD14^++^ CD16^−^), non-classical or patrolling (CD14^+^ CD16^++^) and intermediate (CD14^++^ CD16^+^). Classical monocytes, the largest subset, express the chemokine receptor CCR2, which mediates recruitment to sites of inflammation, where monocytes can differentiate *in situ* to macrophages or dendritic cell populations. Non-classical monocytes “patrol” the blood vessels to remove damaged cells and debris and resolve inflammation in damaged tissues [reviewed in (4)]. In mice, subsets are identified by Ly6C and CD11 markers (implicated adhesive interactions). Ly6C^hi^ monocytes resemble the classical and intermediate human monocytes, and Ly6C^low^ monocytes are similar to human non-classical monocytes. Human and mouse monocyte subsets play similar roles in host defense (5). In this rough guide, we summarize important recent discoveries related to the role of monocytes in innate immunity to malaria. For a summary of older literature, the reader is referred to Chua et al. (6).

## Roles of Monocytes

### Phagocytosis

**Figure 1:**
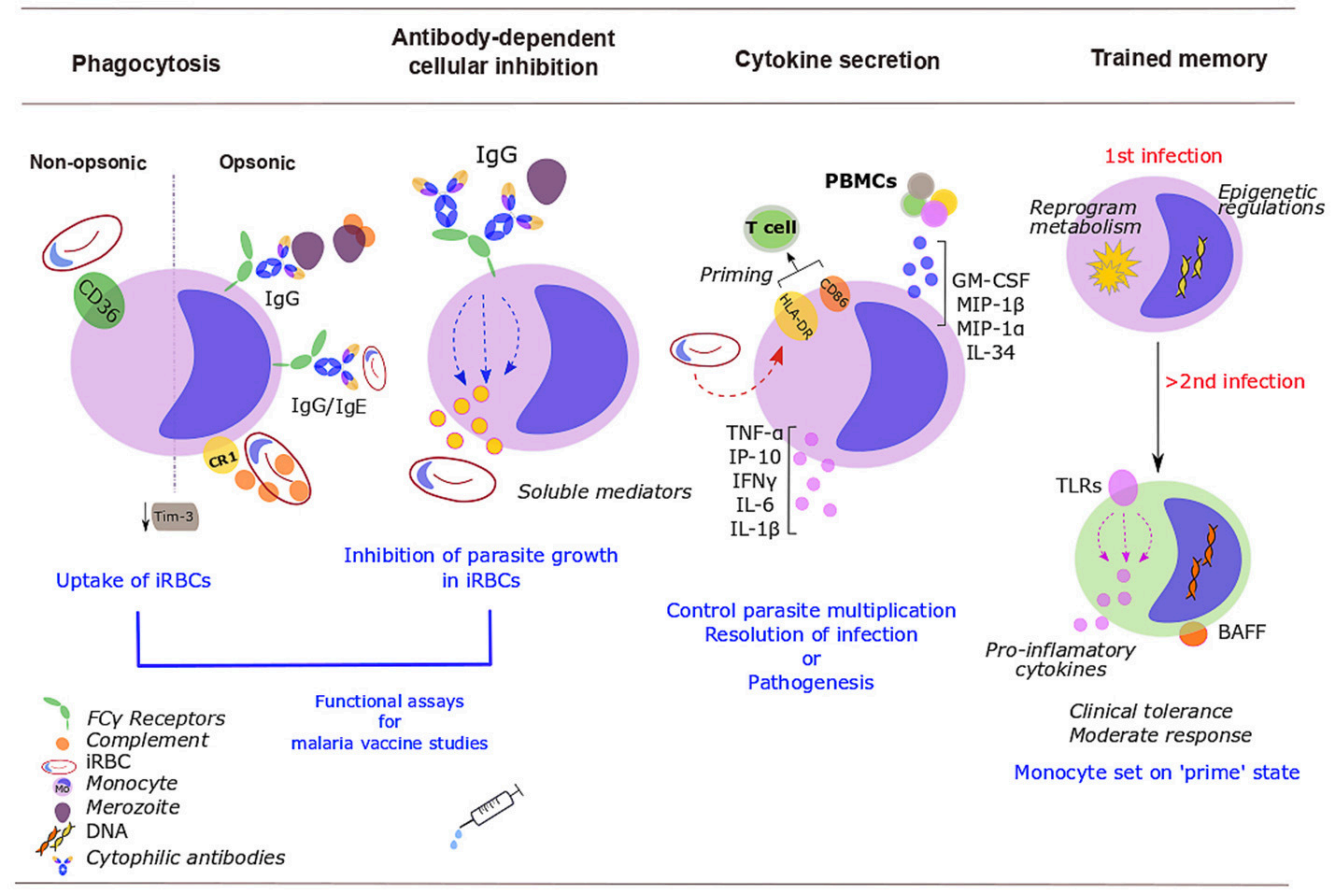
Roles of monocytes during human malaria infection. Monocytes control parasite burden and contribute to host protection (or pathogenesis) through several mechanisms. Infected red blood cells (iRBCs) and merozoites are removed via opsonic or non-opsonic phagocytosis. Opsonic phagocytosis is mediated by either complement [binds to complement receptor 1 (CR1)] or malaria-specific antibodies (bind to Fcγ-receptors). Non-opsonic phagocytosis largely relies on CD36. Malaria down-regulates mucin-domain-containing molecule 3 (Tim-3). Soluble mediators released upon exposure to cytophilic antibodies stop *Plasmodium* from growing inside iRBCs [antibody-dependent cellular inhibition (ADCI)]. Monocyte phagocytosis and ADCI correlate with protection and might be used *in vitro* in malaria vaccine studies. Cytokine production balances protection/ susceptibility in the host. *Plasmodium* iRBCs increase HLA-DR; expression of activation markers, HLA-DR and CD86, might prime T cells response. PBMCs further activate and recruit monocytes through increased production of GM-CSF, MIP-1β, IL-34, TNF-α, or MIP-1α. Upon stimulation with *P. falciparum* iRBCs, monocytes secrete TNF-α, IL-1β, IL-6, IP-10, and IFN- γ. After a challenge with *Plasmodium*, monocytes develop some sort of memory. Monocytes undergo epigenetic modifications, metabolic rewiring and altered cytokine secretion. These changes “prime” monocytes to a more moderate response to secondary encounters with the parasite. Some of these changes will persist over time, including the expression of Toll-like receptors (TLRs) (involved in inflammatory cytokine production) and the membrane-bound form of the B-cell activating factor (BAFF).

Monocytes appear not to phagocytose RBCs infected with mature gametocytes, the sexual erythrocytic stage that transmits to the mosquito (7), but their ability to phagocytose merozoites and asexual iRBCs is pivotal to control of parasitemia (Figure 1). Antibodies are not essential for phagocytosis, but *Plasmodium*-specific IgGs enhance the phagocytic activity of monocytes and this correlates with protection and reduces the risk of symptomatic malaria (8–12). On the other hand, *Plasmodium*-specific IgEs and activated monocytes have a role in disease severity (11). The intermediate CD14^++^ CD16^+^ monocytes were the most efficient subset at phagocytosis of *Plasmodium vivax* iRBCs (which correlated with their expression of the adhesion molecules ICAM-1 and PECAM-1) (13) and IgG or complement opsonised *P. falciparum* iRBCs (10). With increasing age and malaria exposure, individuals develop protective IgGs to surface antigens of iRBCs, particularly to *P. falciparum* erythrocyte membrane protein 1 (PfEMP1) (8). Antibodies to merozoite surface proteins (MSPs) correlate with protection too. Opsonizing antibodies against MSP1 can recruit monocytes for merozoite phagocytosis (14), while cytophilic immunoglobulins (IgG1 and IgG3) against MSP2 and MSP3 strongly activate monocytes (15). Bergmann-Leitner reported that the relative phagocytic activity of monocytes *in vitro* (defined as “opsonization index”) serves as a surrogate marker of protection induced by the RTS,S/AS01 vaccine. Surprisingly, they found that protected subjects showed lower opsonization efficiency (16). Likewise, non-opsonic phagocytosis, which largely relies on scavenger receptor CD36 (17), plays a role in removal of iRBCs, and might be particularly relevant in conditions in which antibody responses are compromised such as HIV infection. HIV infection additionally impairs monocyte functions, including non-opsonic phagocytosis of iRBCs (18). Malaria parasites also modulate monocyte protein expression. For example, iRBCs inhibit monocyte surface expression of complement receptor 1 (CR1 or CD35), and thus impair phagocytosis of circulating immune complexes that can bind to active C3b and C4b, potentially contributing to inflammatory pathology in malaria (19). Additionally the T-cell immunoglobulin- and mucin-domain-containing molecule 3 (Tim-3) that inhibits phagocytosis is down-regulated in monocytes during malaria infection (20).

### Antibody-Dependent Cellular Inhibition

Antibody-dependent cellular inhibition (ADCI) is a major mechanism of defense in acquired immunity to malaria (21), in which monocytes, upon exposure to merozoites that have been opsonized with cytophilic antibodies [IgG1 and IgG3] subtypes to merozoite surface antigens (22), release soluble mediators that inhibit the growth of parasites in iRBCs (21, 23) (Figure 1). ADCI assay performed *in vitro* correlates with clinical protection from malaria and has been proposed as a functional assay for malaria vaccine studies (24). It has been used to assess the potential of merozoite antigens as vaccine candidates (25).

### Cytokine Secretion

Following infection with *Plasmodium*, early secretion of pro-inflammatory cytokines by monocyte lineage cells helps to control parasite multiplication and resolution of infection, but excessive production contributes to pathogenesis. In humans, monocytes are an important source of these early cytokine responses (Figure 1).

Differences in activation and persistence of monocytic lineage cells between symptomatic and asymptomatic infection were reported in Haitian adults and might be due to higher production of GM-CSF, MIP-1β, or IL-34 upon exposure to *P. falciparum* schizont lysate from the PBMCs of these groups. These cytokines are likely drivers of a non-sterilizing immunity with lower parasite loads (premunition), by attracting and potentiating viability, opsonic phagocytosis and cytokine secretion in monocytes (26). In acute uncomplicated *P. falciparum* malaria in children, monocytes increase secretion of the proinflammatory cytokines TNF-α, IP-10 (CXCL10), IFN-γ, and IL-6 and decrease phagocytosis of iRBCs (27). In acute *P. vivax* infection, inflammatory mediators, TNF-α, IL-6, and IL-8, are primarily secreted by inflammatory and classical monocytes (28). TNF-α and IFN-γ influence the sequestration of iRBCs and activation of the endothelia by upregulating ICAM-1 and other adhesion molecules (28).

During blood-stage infection with *P. falciparum*, human inflammatory monocytes from naïve adults upregulate the expression of activation markers HLA-DR and CD86, which are associated with priming of T cells (29). In severe malaria in Malawian children, the inflammatory monocyte subset was expanded and activated with higher plasma levels of inflammatory cytokines (IFNα, IFNγ, TNF-α, IL-6) and chemokines (CCL2, CCL3, CCL4, CXCL10) than in convalescence (30). In another study from Malawi, monocytes from children with severe malaria had lowered expression of the activation markers CD18, HLA-DR, and CD86. compared to healthy controls (31). When whole blood was stimulated *in vitro* with LPS, monocytes from children with severe malaria produced less proinflammatory cytokines TNF-α and IL-6 than cells from healthy controls. Exposure to *P. falciparum* iRBCs also alters monocyte activation. It resulted in upregulation of HLA-DR expression on naïve monocytes derived from haematopoietic stem cells (32). Monocytes from PBMCs of Papua New Guinean children with severe malaria responded *in vitro* to *P. falciparum* iRBCs by secreting higher quantities of TNF-α, MIP-1β, and MIP-1α (implicated in monocyte activation and recruitment) than healthy children or children with uncomplicated malaria (33).

## Spleen

During the asexual stage, *P. falciparum* iRBCs become more rigid, and are retained by mechanical filtration in the spleen (34), which has a pivotal role in the immune response against malaria infection (35), reduction of parasitemia and clearance of infection (36).

In mice, upon infection with *P. chabaudi*, monocytes egress from the bone marrow and migrate to the spleen, reducing blood stage parasitemia by phagocytosing iRBCs and producing reactive oxygen intermediates (37). In deceased Malawian children, dysfunction in the ability of the spleen to phagocytose parasites has been linked with higher parasite loads and a more rapid progression to death (36).

Although the spleen does not contribute to the pool of circulating monocytes during *P. falciparum* infection in non-human primates (38), local splenic inflammatory monocytes could play various roles; *in situ*, murine splenic monocytes/macrophages stimulated by IFN regulatory factor 3 (IRF3) (39) coach activated CD4^+^ T cells toward a protective Th1 fate during infection with blood-stage *Plasmodium* parasites (39, 40). Murine splenic monocytes also migrate to the brain as CCR5^+^CXCL9/10^+^ MO-DCs inducing neuroinflammation (41). Little work has been done on human spleen; differences and similarities in the pathological changes observed in the spleens of human and mice during *Plasmodium* infection are discussed in Urban et al. (42).

## Role of Monocytes in Clinical Manifestations

### Severe Malaria Anemia

Severe malaria anemia (SMA) [hemoglobin <5.0 g/dL], is the most common severe manifestation of malaria in young children and pregnant women. SMA is caused by sequestration of RBCs in the spleen, loss of both RBCs and iRBCs and suppression of erythropoiesis and abnormal development of RBCs precursors (dyserythropoiesis) in the bone marrow (43). During SMA, monocytes contribute to SMA through phagocytosis and secretion of pro-inflammatory cytokines (43). Past studies found a correlation between monocytes loaded with *P. falciparum* hemozoin (a parasite by-product) and suppression of erythropoiesis; while high levels of these monocytes predicted SMA (44). More recent studies in non-human primate macaque models for *P. vivax* infection showed that during acute malaria, monocytes loaded with hemozoin suppress erythropoiesis in the bone marrow by inducing apoptosis of the erythroid progenitors via IFNγ and antagonization of GATA1 transcriptional networks (45).

### Cerebral Malaria

Cerebral malaria (CM) is the most life-threatening presentation of *P. falciparum* malaria in young African children. Impaired consciousness, delirium or coma may be accompanied with swelling of the brain, intracranial hypertension or changes in the retina (46). Sequestered iRBCs block intracerebral blood vessels, and infiltrating immune cells including monocytes accumulate in the same vessels and secrete inflammatory cytokines (47); monocyte accumulation is greater in HIV-infected CM children (48).

During experimental cerebral malaria (ECM) in *P. berghei* ANKA-infected mice, Ly6C^hi^ monocytes are the main sequestered leukocyte population, inducing inflammation and disease (49), and aggravating brain inflammation by recruiting CD4^+^ and CD8^+^ T cells (49, 50) and by secreting the chemokine CXCL10 (50, 51). CXCL10 mediates cerebral adhesion and accumulation of T cells, driving the onset of CM (51). In *P. berghei* ANKA-infected mice also, *Plasmodium* specific CD8^+^ T cells regulate the adhesion and rolling behavior of monocytes (49). Other molecules that might mediate monocyte accumulation in *P. berghei* ANKA model include increased C5a (28) and inhibition of nitric oxide activity (52) and the chemokine MCP-1/CCL2 (53).

At present, there is no clear consensus on the extent to which findings in mice models translate to humans, given data suggesting that *P. berghei* ECM is primarily driven by leukocyte accumulation, whereas human CM is principally due to iRBC sequestration in the cerebral vasculature (54).

### Placental Malaria

Malaria in pregnant women can restrict fetal growth or result in premature delivery and elevates the risk of maternal anemia and infant mortality. Infection is initiated by the sequestration of iRBCs that bind to the chondroitin sulfate A expressed on the placenta through VAR2CSA, a member of the PfEMP1 protein family (55, 56). Subsequently, circulating monocytes and tissue-resident macrophages accumulate in the intervillous space of *P. falciparum*-infected placentas initiating a local inflammation (intervillositis), a major determinant of the severity of the disease and intrauterine growth restriction (57).

Recently, Aubouy et al. described a protective role for monocytes and macrophages during pregnancy (58). The expression of the scavenger receptor CD36 and Heme-Oxygenase-1 (HO-1) on circulating monocytes correlated with the levels of anti-inflammatory markers IL-10 and CD163, and with an increase in infant birth weight (58). These findings diverge from other works that link high levels of IL-10 in both plasma and placenta with low birth weight babies and parasitemia (59). In addition, high numbers of circulating monocytes and high plasma IL-10 concentrations predict maternal anemia at delivery (60) although how these two factors entangle is unknown. The membrane receptor CD163 contributes to the anti-inflammatory response by scavenging hemoglobin: haptoglobin complexes, resolving monocyte activation and improving clinical outcome (58). Levels of soluble sCD163 shed by monocytes correlate negatively with birth weight and maternal hemoglobin levels (61).

Monocyte opsonic phagocytosis of iRBCs is also an important component of the acquired immune response in pregnancy malaria. Multigravid women generate protective IgG antibodies to VAR2CSA, (9) and antibodies recognizing domains DBL5 and DBL3 are effective inductors of monocyte phagocytosis (12). Limited cross-reactivity between isolates may hinder the development of a vaccine (12).

### Acute Lung Injury

Children and adults experiencing severe malaria are prone to develop acute lung injury (ALI) and its most severe form, acute respiratory distress (ARDS). Patients with ARDS display dyspnoea, cough, and chest tightness and can develop hypoxia leading to death. These complications arise probably from increased alveolar capillary permeability, triggered by iRBC sequestration in pulmonary vasculature and secondary local inflammation followed by cytokine secretion. Bronchial IL-33 might be a driver of pulmonary edema in human patients since it positively correlates with CD68^+^ monocyte accumulation (62).

In mice, most leukocytes in the pulmonary interstitium are bone-marrow derived inflammatory monocytes (63) and macrophages (64). In this model, although monocytes prevent tissue damage by CD36-mediated non-opsonic phagocytosis of iRBCs (63), they might also contribute to the inflammatory manifestations of ARDS. The β_2_ leukocyte integrin, α_D_β_2_ (CD11d/CD18) increases alveolar-capillary membrane permeability, the accumulation of monocyte and macrophages, and lung edema (64), while another β_2_ integrin, α_M_β_2_ (CD11b/CD18) is important for parasite clearance during ALI (65). In humans, post-mortem histology reveals monocyte accumulation in pulmonary vessels (47), which could also explain the impaired gas transfer observed in adults with uncomplicated malaria (66).

**Figure 2:**
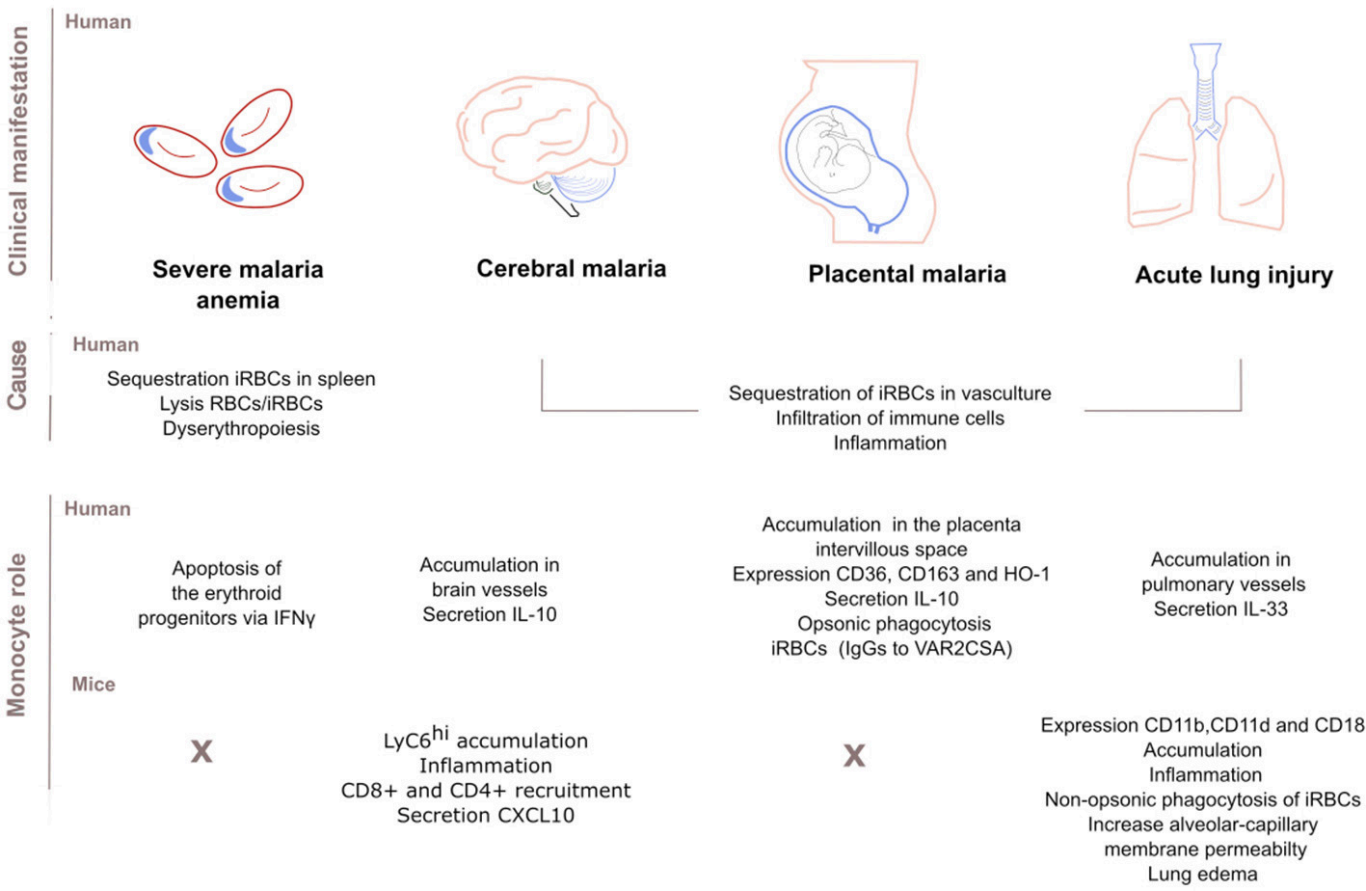
Role of monocytes in the clinical manifestations of malaria. Sequestration of infected red blood cells (iRBCs) in the vasculature of main organs (spleen, brain, placenta, or lungs) is associated with severe disease. Infiltration of immune cells and subsequent inflammation also contributes to pathogenesis. Dyserythropoiesis and lysis of uninfected RBCs and iRBCs cause severe malaria anemia too. Human monocytes infiltrate and accumulate in the vessels of those main organs. There, monocytes secrete anti- or pro-inflammatory cytokines, express surface markers or phagocytose IgG-opsonised iRBCs. Studies disagree over whether these mechanisms drive host protection or susceptibility. Similarly, mouse monocytes accumulate and drive inflammation in the brain and lung. In the mouse model of cerebral malaria, monocytes further recruit CD8^+^ and CD4^+^ cells by secreting the chemokine CXCL10. In the lung, monocyte CD11b/CD18 expression is important for parasite clearance while the integrin CD11d/CD18 expression increases permeability of the alveolar-capillary and causes lung edema. There is no clear consensus on to what extent findings in mice models of malaria infection translate to humans but human and mouse monocyte subsets play similar roles in host defense.

In summary, severe forms of malaria are linked to the ability of iRBCs to sequester in the vasculature of organs like lungs, placenta, brain, or spleen. After iRBC sequestration, immune cells including monocytes may accumulate in the vasculature. Local monocytes become an immune hub by removing merozoites or iRBCs, inhibiting parasite growth, secreting cytokines or recruiting other cells of the immune system; such activities might improve or worsen the progress of clinical manifestations. Some of the roles of monocytes in severe disease are illustrated in Figure 2.

## Effect of Malaria Parasites on Monocyte Functions

Beyond the effect of iRBCs sequestration and lysis, hemozoin, parasite DNA or secreted vesicles also contribute to malaria pathogenesis. *Plasmodium* digests hemoglobin within RBCs. The metabolic by-product, heme, is polymerised into the crystal structure hemozoin (HZ) (67). Circulating and resident monocytes phagocytose and accumulate HZ. The proportion of circulating HZ-containing monocytes increases during malaria (31) and this correlates with disease severity (68). Particularly, HZ-containing monocytes are significantly elevated in patients with SMA (69), and HZ appears to be important in the induction of dyserythropoiesis and apoptosis in nascent erythroid cells (69). Overall, ingested HZ weakens the immune system by destroying monocytes (70), impeding their maturation to dendritic cells (71) or impairing their overall functionality (72).

HZ influences monocyte function in a number of ways. It exacerbates the production of pro-inflammatory markers such as IL-1β and TNF-α (73). This may be due to the association between HZ crystals and the lipid 15-HETE (15-hydroxy-eicosatetraenoic acid), which upregulates expression and release of matrix-metalloproteinase 9 (MMP-9), in turn implicated in the secretion of inflammatory cytokines (74). HZ also induces monocyte dependent expression and secretion of TIMP-1, the endogenous inhibitor of MMP-9 (75), although the role of TIMP-1 in malaria pathogenesis remains unknown. HZ lowers monocyte expression of adhesion molecules (CD11b, CD11c, and CD18) (31), and diminishes *in vitro* monocyte diapedesis and chemotactic motility toward MCP-1, TNF-α, and FMLP (formyl-methionyl-leucyl-phenylalanine), partially explaining patients' immunosuppression (31). In mice, pulmonary HZ is associated with the recruitment of inflammatory cells, including inflammatory monocytes (76).

From within RBCs, *Plasmodium* communicates with other cells by releasing vesicles to the extracellular milieu. Monocytes internalize these extracellular vesicles (EVs) (77). EVs from erythrocytes infected with ring-stages of *P. falciparum* modify the functionality of human monocytes, in part by upregulating antigen presentation pathways and enhancing the interferon response (78), although EVs containing PfEMP1 downregulate “defense response” pathways (78), consistent with observations that PfEMP1 suppresses the immune response by dampening monocyte inflammatory cytokine and chemokine release (79). Vesicles may also deliver non-coding parasite RNAs and gDNA to the monocyte (77). Once inside, specific cytosolic sensors detect *Plasmodium* DNA, triggering the transcription of type I IFN genes by the stimulator of TNF genes (STING) pathway (77). Interestingly, the ingestion of circulating DNA-containing immunocomplexes (ICs) is an alternative way for parasite's DNA to gain access to the monocyte cytosol (80). In this case, ICs induce the assembly of the NLRP3/ASC^+^ and AIM2/ASC^+^ inflammasomes, activation of caspase-1 and secretion of IL-1β (80). In parallel, DNA bound to HZ leads also to caspase-1 dependent IL-1β secretion but through NLRP12 and NLRP3 inflammasome (81). More importantly, inflammasome assembly induces a “primed” state in monocytes which is partially dependent on TLR9 activation, and when exposed to a second microbial challenge these cells produce deleterious amounts of IL-1β (81).

## Effect of Malaria Parasites on Circulating Monocyte Counts

As the disease progresses, malaria alters blood cell counts. This might correlate with the ability of the individual to mount a proper immune response and reflect different levels of immunity to malaria (82). As such, changes in leukocyte numbers and cytokine profiles have been assessed as markers for the course of the infection and the immune response (83). In malaria- naïve volunteers, during the liver stage of *P. falciparum*, neutrophil, lymphocyte and monocyte counts increase (84). This is consistent with other studies reporting an increase in CD14^+^ cells in primary *P. falciparum* infection (20) and an expansion of the inflammatory intermediate CD14^++^CD16^+^ monocyte subset during uncomplicated *P. falciparum* malaria in children (27). In contrast, both increased (13) or decreased (85) numbers of circulating monocytes have been observed in patients during *P. vivax* infection.

Leukocyte ratios might constitute surrogate markers for immunity. The monocyte to neutrophil ratio has been associated with severe malaria, especially in semi-immune patients (82). If low, this ratio may indicate a risk for developing complicated malaria (86). By contrast, a high monocyte to lymphocyte ratio (MLCR) better discriminates between clinical malaria and controls (82), correlating with increased risk for clinical malaria (87). Most importantly, variation in RTS,S vaccine efficacy between individuals is significantly predicted by differences in the MLCR ratio (88).

Some studies report associations between high circulating monocyte counts and high parasitemia (85), but others report that monocyte counts are significantly lower in patients with high parasitemia (89). This disagreement might be due to differences in the hematological profile of circulating cells between geographical areas (90). Regardless of the number of malaria episodes experienced, age and season also affect hematological indices and white blood cell subsets, including the monocyte count (91). Still, it is possible to establish reference intervals for hematological parameters that are comparable and applicable across areas with similar transmission conditions (92). Leukocytes also undergo changes in volume, conductivity and light scatter that reflect changes in function in different types of infections (93). In clinical malaria, monocytes increase their volume and relative quantity (94) while their internal composition (conductivity) significantly differs from that observed in non-malaria fevers (93).

## Trained Immunity in Monocytes

Vaccines target the adaptive response, but clinical and epidemiological data prove that vaccines such as BCG exert nonspecific effects too (95). Possible mechanisms included “heterologous immunity,” driven by cross-reactive T-lymphocytes; or trained memory in innate immune cells. After a first stimulus, the “trained immunity” phenotype relates to a “prime” state that enhances reactivity of monocytes/macrophages or NK cells to a secondary challenge (96). This phenotype involves epigenetic modifications, metabolic rewiring or cytokine secretion. This may well be important in malaria, where each new infection with *Plasmodium* activates the innate response (27).

Reinfections with *Plasmodium* can alter monocyte metabolism, chromatin, receptors expressed or the frequencies of each subset. But these alterations may either lower (tolerance) or increase host resistance (trained immunity) to reinfections (75) (Figure 1).

Compared to vivax-naïve individuals, semi-immune people reprogram their myeloid cells' metabolism to a more coordinated response which could influence clinical tolerance to reinfections and result in asymptomatic infections with *P. vivax* (97). This acquired tolerance moderates the immune response to *P. vivax* infection, as observed in gene transcription profiles in peripheral blood comparing semi-immune to malaria-naïve individuals (98).

The largest nomadic ethnic group in Africa, the Fulani, are more resistant to *P. falciparum* than their geographical counterparts (99). Global transcriptional and DNA methylation analysis of the whole blood show that the chromatin in Fulani people's monocytes (and no other cell) is set on a prime state; thus upon *P. falciparum* infection, epigenetic regulations in monocytes induce an enhanced pro-inflammatory response. Compared to a sympatric group, Fulani adults show higher levels of inflammasome activation, and in the presence of malaria infection this translated into higher secretion of IL-1β and IL-18 (99).

It is thought that pattern recognition receptors including TLRs could assist monocytes to mount some sort of memory specific to individual organisms, including *Plasmodium* (100). Children with severe malaria show a lowered expression of TLR2 and TLR4 which correlates with monocyte inactivation and reduced inflammatory cytokine production (31). In the acute phase of infection, monocytes overexpress genes involved in TLR signaling (TLR8, LY96, MYD88) (27). More importantly, these changes persist in convalescence when compared to monocytes from healthy malaria-naive controls (27). Another long-lasting effect is the increased expression on various monocyte subsets of the membrane-bound form of B-cell activating factor (BAFF), essential in B-cell homeostasis (101).

History of exposure also influences the relative number of circulating monocyte sub-populations (102). In malaria-naive individuals, frequencies of classical and intermediate monocyte sub-populations expand during blood stage infection with *P. falciparum* (29). In Kenyan children, after recovery from acute uncomplicated *P. falciparum* malaria, the inflammatory “intermediate” subset stops its expansion and returns to levels of healthy asymptomatic children (27).

## Future Directions

Caution must be taken when interpreting data regarding monocytes' roles in malaria infection. Monocytes are an heterogenous population of cells whose functionality is further shaped by host age, geography and history of exposure (31). This is consistent with training or tolerance effects that could explain the *contradictory behavior* of monocytes observed across different settings, independent of differences in protocols and analysis. To minimize these discrepancies, Udomsangpetch et al. have developed a model of mononuclear cells generated from hematopoietic stem cells, that evaluates *in vitro* the interaction between naïve immune cells and malaria parasites (32). Whole genome association studies (GWAS) might be used as a tool to identify genetic differences that can further explain why monocytes respond differently across geographical areas (103, 104). Other inconsistencies, like responses to malaria vaccines reported to date, may in part be attributed to variations in the monocyte response (75), influenced by the adjuvant used and the age of the patient (105).

As we saw in this *rough guide*, the extraction of monocytes from peripheral blood is a common method to study their response in isolation. However, it will be important to consider analyses that integrate third players in the interaction between monocytes and parasites. Gene expression profiling of whole blood might be used to identify the type and duration of the immune response in infection (98). But as Zak et al. point out, innate responses in the periphery might not reflect what happens locally: monocyte re-localization to an inflammatory site could explain why a gene is less present or transcribed in blood (106). In this regard, systems vaccinology offers a powerful approach quantifying innate and adaptive responses in different compartments (106).

## Author Contributions

AO-P gathered all the papers included in the review, drafted the manuscript and designed (Figures 1, 2). SR corrected and revised it critically for important intellectual content, SR also gave final approval for the version submitted.

### Conflict of Interest Statement

The authors declare that the research was conducted in the absence of any commercial or financial relationships that could be construed as a potential conflict of interest.
